# The long-term risk of cardiovascular disease among women with a history of hypertensive disorders of pregnancy: a systematic review of clinical practice guidelines

**DOI:** 10.1186/s12872-023-03446-x

**Published:** 2023-09-09

**Authors:** Jessica Atkinson, Grace Simpson, Susan P Walker, Stephen Tong, Roxanne Hastie, Anthea Lindquist

**Affiliations:** 1 Department of Obstetrics and Gynaecology, University of Melbourne, Mercy Hospital for Women, Heidelberg, VIC 3084 Australia; 2 Mercy Perinatal. Mercy Hospital for Women, Heidelberg, VIC 3084 Australia

**Keywords:** Cardiovascular disease, Pre-eclampsia, Gestational hypertension, Postpartum, Risk reduction, Clinical practice guidelines, Pregnancy, Women’s health

## Abstract

**Background:**

The lifelong risks of cardiovascular disease following preeclampsia and gestational hypertension are well-established. However, it is unclear whether this evidence has been translated into clinical practice guidelines. Thus, this review aimed to assess the quality and content of Australian clinical practice guidelines regarding the risk of cardiovascular disease following gestational hypertension and preeclampsia.

**Methods:**

We conducted a systematic search of MEDLINE (Ovid), EMBASE (Ovid), and CINAHL databases, as well as hospital, obstetric society, and medical college websites. Publications were included if: they were a clinical practice guideline; were published in the previous ten years; and included recommendations for the management of future cardiovascular disease risk following hypertensive disorders of pregnancy. Quality assessment was performed using Appraisal of Guidelines for Research and Evaluation Instrument Version Two (AGREE-II) and AGREE Recommendations Excellence Instrument (AGREE-REX).

**Results:**

Eighteen guidelines were identified, and of these, less than half (n = 8) included recommendations for managing future cardiovascular risk following hypertensive disorders of pregnancy. Across these eight, four main counselling recommendations were found regarding (1) risk of future cardiovascular disease; (2) risk factor screening; (3) lifestyle interventions; and (4) prenatal counselling for future pregnancies. The quality and content of these recommendations varied significantly, and the majority of guidelines (87.5%) were assessed as low to moderate quality.

**Conclusions:**

There are limited Australian clinical practice guidelines providing appropriate advice regarding future risk of cardiovascular disease following hypertensive disorders of pregnancy. The quality and content of these guidelines varied significantly. These findings highlight the need for improved translation from evidence-based research to enhance clinical care and guidance.

**Supplementary Information:**

The online version contains supplementary material available at 10.1186/s12872-023-03446-x.

## Introduction

Hypertensive disorders of pregnancy, including preeclampsia and gestational hypertension, affect between 5% and 10% of pregnancies globally [1]. These disorders are characterised by new-onset hypertension during pregnancy, and preeclampsia is further characterised by end-organ damage. They can have devastating consequences during pregnancy for both mother and baby, including serious maternal morbidity, mortality, and stillbirth [2].

Long-term cardiovascular sequelae are also associated with preeclampsia and gestational hypertension and have been well-characterised. Following an affected pregnancy, women are at increased risk of chronic hypertension, heart failure, coronary heart disease, and death due to cardiovascular disease, compared with their unaffected peers [3–9]. These risks are not remote, and often manifest within a few years following pregnancy [10–12]. However, recent research has shown that many women are unaware of their risk [13, 14].

It is vital that women are counselled about risk mitigation either during pregnancy or early postpartum. Lifestyle interventions and regular risk factor monitoring (of blood pressure, glucose, and cholesterol) have been shown to reduce the risk of cardiovascular disease among the general population, and trials are underway to validate these interventions among postpartum women [15–21].

The Heart Foundation Australia recommends that women with a history of hypertensive disorders of pregnancy maintain a healthy lifestyle and have regular clinical monitoring to reduce their risk of cardiovascular disease [22]. However, it is unclear whether these recommendations have been translated to obstetric practice guidelines. Women affected by hypertensive disorders of pregnancy have been reported to be more motivated to engage in sustained lifestyle modification if they are first approached during their pregnancy or early postpartum [23, 24]. Pregnancy can thus be viewed as an opportunity to engage with women and initiate positive lifestyle modifications.

Clinical practice guidelines reflect current evidence and expert recommendations for best practice and clinical care [25]. Various guidelines targeted towards obstetric care providers exist regarding the detection and management of hypertensive disorders of pregnancy; however, it is unclear whether they adequately address the future risk of cardiovascular disease. Australian research has demonstrated that there is a significant gap in the understanding of future risk after an affected pregnancy, not only among affected women, but also among relevant healthcare providers [26]. It has been shown that women prefer to be informed about their future risk of disease from their healthcare provider, rather than sourcing information through other channels [27]. Given this, it is critical that appropriate recommendations for managing future risk of cardiovascular disease following hypertensive disorders of pregnancy are included in guidelines for healthcare providers, so that these recommendations can reach affected women.

Thus, this review aimed to assess the quality and content of current Australian clinical practice guidelines relating to the long-term management of cardiovascular disease among women affected by hypertensive disorders of pregnancy.

## Methods

This review was conducted according to the Preferred Reporting Items for Systematic Reviews and Meta-Analyses (PRISMA) guidelines (Supplementary Table 1) [28]. A protocol was prospectively developed and registered with PROSPERO (CRD42022328892). Ethical approval was not required as this was a review of published material.

### Identification and selection of guidelines and recommendations

We included guidelines published in Australia during the preceding ten years (1 January 2012–23 May 2022) that were intended for screening, diagnosis, or management of *de novo* hypertensive disorders of pregnancy (Supplementary Tables 2–4). *De novo* hypertensive disorders of pregnancy included gestational hypertension and preeclampsia, as defined by the International Society for the Study of Hypertension in Pregnancy [29]. Pre-existing hypertensive disorders (such as chronic hypertension) were not included as management of these conditions differs to that of hypertensive disorders which develop during pregnancy. Only guidelines intended for use by healthcare professionals were included. Guidelines were later excluded if they did not include recommendations pertaining to the prevention or management of future cardiovascular disease among affected women.

A search of online databases MEDLINE (Ovid), EMBASE (Ovid), and CINAHL was conducted on 23 May 2022 (Supplementary Tables 2–4). Australian government, tertiary maternity hospital, obstetric society, and medical college websites were also screened for relevant guidelines. Reference lists of included guidelines were reviewed for additional relevant materials. Only guidelines available in English were reviewed.

**Fig. 1:**
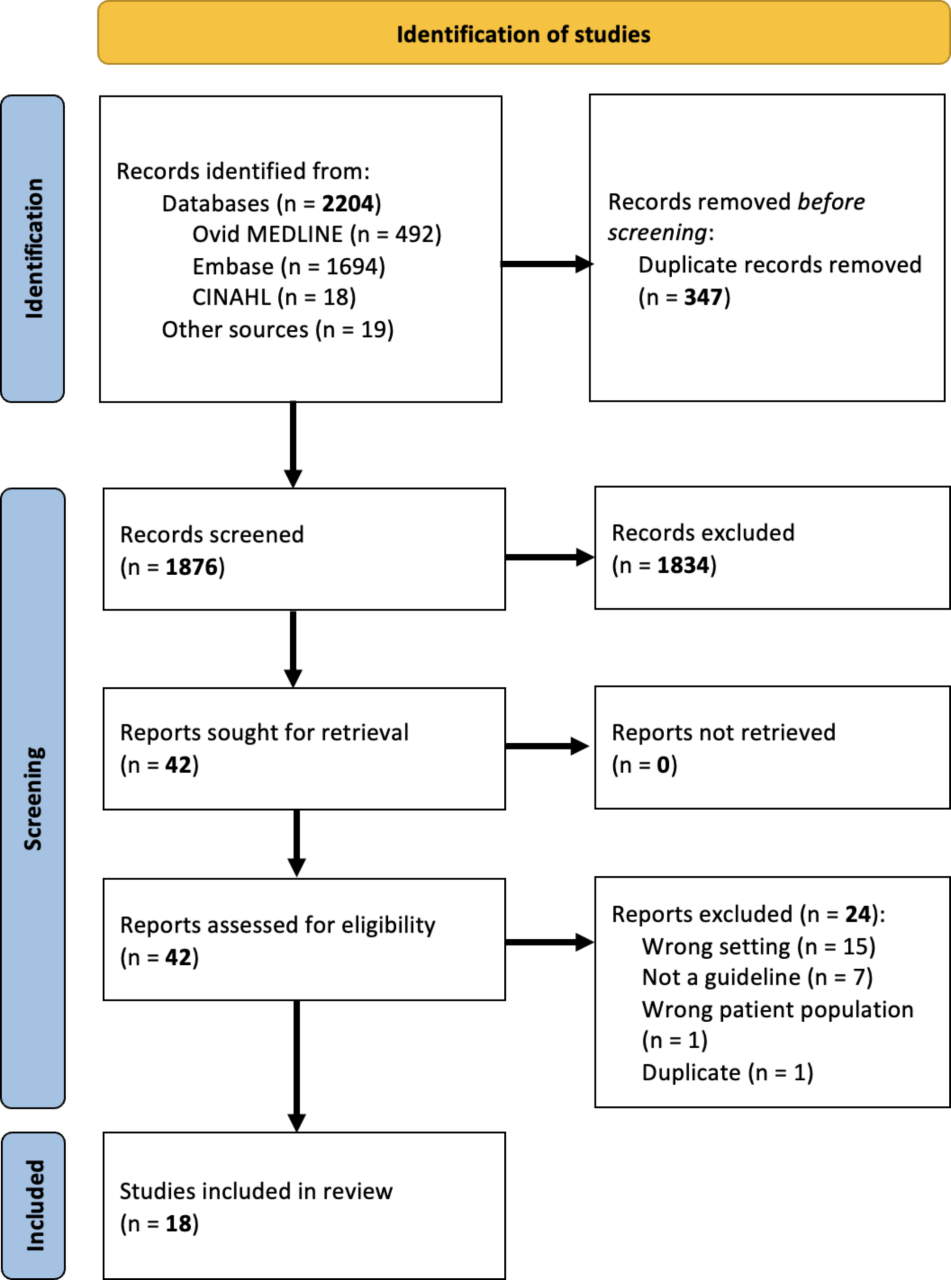
Preferred Reporting Items for Systematic Reviews and Meta-Analyses (PRISMA) diagram. Adapted from: Page et al. (2020) [28].

Title, abstract, and full-text screening was performed by two reviewers (JA, GS) (Fig. 1). The results of screening at each stage were compared and discrepancies were settled by screening by a third reviewer (AL). Data extraction was performed by two reviewers (JA, GS), and any discrepancies were settled by reviewer discussion or consultation with a third reviewer (AL). This process was facilitated by Covidence online software [30].

The following fields were extracted from each included guideline: title; publishing institution; authors; year of publication; jurisdiction; population addressed; whether the guideline was adapted from previous material; and the content of specific recommendations pertaining to postpartum counselling or management of future cardiovascular disease (Tables 1 and 2).

**Table 1:** Characteristics of guidelines with recommendations for postpartum management of cardiovascular disease risk

Guideline	Institution	Author(s)	Jurisdiction	Adapted from previous guideline	Population addressed
Guideline for the management of hypertensive disorders of pregnancy (2014) [34]	Society of Obstetric Medicine Australia and New Zealand (SOMANZ)	Lowe SA, Bowyer L, Lust K, McMahon LP, Morton M, North RA, Paech M, Said JM	Multi-National (Australia and New Zealand)	No	Women with hypertensive disorders of pregnancy in Australia and New Zealand
Hypertensive Disorders in Pregnancy TEHD Maternity Guideline (2019) [41]	Northern Territory Government/Top End Health Services	Powell V	Territory-Based (Northern Territory)	Yes – Adapted with permission from Queensland Health [43]	Women with hypertensive disorders of pregnancy
Hypertensive Disorders of Pregnancy (2020) [47]	South Australia Health	-	State-Based (South Australia)	Yes – Adapted from SOMANZ guideline and material from the Royal Australian College of Obstetricians and Gynaecologists (RANZCOG)	Women with hypertension in pregnancy
Hypertension and Pregnancy (2021) [43]	Queensland Health	-	State-Based (Queensland)	No	Women with hypertension in pregnancy
Hypertension – Management in pregnancy (2020) [44]	Royal Hospital for Women (NSW)	-	Hospital-Based (Royal Hospital for Women, New South Wales)	No	Women with hypertension in pregnancy
Hypertensive disorders in pregnancy, pre-eclampsia, eclampsia clinical guideline (2022) [39]	Monash Health (VIC)	Rolnik DL, Papacostas K, in collaboration with Maternity Guideline Development Group, Anaesthetics, and Pharmacy	Hospital-Based (Monash Health, Victoria)	Yes – Adapted from SOMANZ guideline	Pregnant or postpartum women who present with, or develop, hypertensive disorders, preeclampsia, and/or eclampsia
Hypertension in Pregnancy (2018) [38]	Liverpool Hospital (NSW)	-	Hospital-Based (Liverpool Hospital, New South Wales)	Yes – Adapted from 2011 NSW Health Guideline	Women with hypertension in pregnancy
Pre-Eclampsia: Management (2020) [49]	The Royal Women’s Hospital (VIC)	-	Hospital Based (The Royal Women’s Hospital, Victoria)	No	Women with preeclampsia

### Quality appraisal of guidelines

Quality assessment was conducted by two reviewers (JA, GS), with discrepancies settled by reviewer discussion or consultation with a third reviewer (AL). Guidelines were assessed using the Appraisal of Guidelines for Research and Evaluation Instrument version two (AGREE II) [31]. The AGREE II tool assesses the quality of guidelines across six domains: scope and purpose; stakeholder involvement; rigour of development; clarity of presentation; applicability; and editorial independence [31].

Quality assessment was also performed for relevant individual recommendations, which addressed postpartum cardiovascular health. Recommendations were assessed using the AGREE Recommendations Excellence Instrument (AGREE-REX) [32]. The AGREE-REX tool complements the AGREE II tool and is used to assess the quality of guideline recommendations by assessing them across three domains: clinical applicability; values and preferences; and implementability [32].

For both the AGREE II and AGREE-REX tools, guidelines are given a percentage quality score for each domain based on assessment criteria [31, 32]. For each domain, a score of < 30% was considered low quality, 30–70% moderate quality, and > 70% high quality. Quality appraisal data was visualised using the robvis risk of bias assessment figure [33].

### Synthesis of guideline recommendations

Data were grouped into national, state-based, and individual hospital-based guidelines. Data regarding guideline recommendations were summarised and reported.

## Results

Our search identified 2,223 papers. After removing 347 duplicates, 1,876 papers underwent title and abstract screening; 42 required full-text screening, and 18 were eligible for inclusion in the final review (Fig. 1, *Supplementary Table *5). Of the 18 guidelines [34–50], only eight (44.4%) included recommendations relating to the increased risk of cardiovascular disease following pregnancy [34, 38, 39, 41, 43, 44, 47, 49] (Table 1). For the purpose of subsequent data analysis, only these eight guidelines were considered.

### Guideline characteristics

Only one national guideline was identified, published by the Society of Obstetric Medicine of Australia and New Zealand (SOMANZ) [34]. There were three included state-level guidelines (published by Queensland Health [43], Northern Territory Government/Top End Health Services [41], and South Australia Health [47]). Only four hospitals had relevant, publicly available guidelines (Monash Health, Victoria [39]; The Royal Women’s Hospital, Victoria [49]; Liverpool Hospital New South Wales [38]; and The Royal Hospital for Women, New South Wales [44]).

Four guidelines were stated to be based on previously published material: the Northern Territory guideline [41] was adapted from a previous version of the Queensland guideline [43]; the Liverpool Hospital guideline [38] was based on a 2011 New South Wales state guideline [51]; South Australia [47] and Monash Health [39] were both adapted from the SOMANZ guideline [34].

### Recommendations for postpartum management

#### Counselling about increased risk of chronic disease

Monash Health [39], South Australia [47], Liverpool Hospital [38], and the Royal Hospital for Women (New South Wales [NSW]) [44] recommend that clinicians counsel women about their increased risk of cardiovascular disease. The Royal Hospital for Women (NSW) [44] included the greatest detail for clinicians, stating that, “women who have had preeclampsia or gestational hypertension are at higher risk of cardiovascular disease, stroke, and venous thromboembolism later in life,” (page 9) and recommending that clinicians, “advise women regarding long-term health implications,” (page 7). Monash Health [39] stated that clinicians should counsel women about, “hypertension and cardiovascular disease risks later in life,” (page 12) and South Australia [47] stated that women are, “at increased risk of cardiovascular disease,” (page 6), whilst Liverpool Hospital [38] only noted that women are at, “increased risk of future chronic hypertension,” (page 6) (Table 2).

**Table 2:** Summary of recommendations relating to the management of future cardiovascular disease risk

Institution	Guideline	Counselling about risk of cardiovascular disease recommended?	Medical Risk Factor Screening Recommended?	Lifestyle interventions recommended?	Prenatal Counselling for Future Pregnancies Recommended?
National Guidelines					
Society of Obstetric Medicine Australia and New Zealand (SOMANZ)	Guideline for the management of hypertensive disorders of pregnancy (2014) [34]		Y	Y	Y
**State-based Guidelines**					
Northern Territory Government/Top End Health Services	Hypertensive Disorders in Pregnancy TEHD Maternity Guideline (2019) [41]		Y	Y	Y
South Australia Health	Hypertensive Disorders of Pregnancy (2020) [47]	Y	Y	Y	
Queensland Health	Hypertension and Pregnancy (2021) [43]		Y	Y	Y
**Hospital-based Guidelines**					
Liverpool Hospital (NSW)	Hypertension in Pregnancy (2018) [38]	Y	Y		
Royal Hospital for Women (NSW)	Hypertension – Management in pregnancy (2020) [44]	Y	Y		Y
The Royal Women’s Hospital (VIC)	Pre-Eclampsia: Management (2020) [49]		Y		Y
Monash Health (VIC)	Hypertensive disorders in pregnancy, pre-eclampsia, eclampsia clinical guideline (2022) [39]	Y		Y	Y

SOMANZ [34], Queensland [43], Northern Territory [41], and South Australia [47] also referred to the increased risk of type 2 diabetes associated with hypertensive disorders of pregnancy. None of the hospital-based guidelines included reference to future diabetes risk.

#### Lifestyle modification

SOMANZ [34], Queensland [43], Northern Territory [41], South Australia [47], and Monash Health [39] included recommendations for lifestyle modification to reduce future risk of cardiovascular disease. SOMANZ [34] recommended, “counselling women [to consider] avoiding smoking, maintaining a healthy weight, exercising regularly, and eating a healthy diet,” (page 33). Queensland [43], Northern Territory [41], South Australia [47], and Monash Health [39] included near-identical recommendations. Queensland [43] and Northern Territory [41] additionally advise that clinicians should, “encourage overweight and obese women to attain a healthy [body mass index] for long term health,” (page 28 and 24, respectively) (Table 2).

#### Risk factor screening

All guidelines except for Monash Health [39] recommended risk factor screening post-pregnancy. These generally include annual blood pressure monitoring (recommended by SOMANZ [34], Queensland [43], Northern Territory [41], South Australia [47], and Royal Hospital for Women [NSW] [44]). The Royal Women’s Hospital (Victoria) [49] and Liverpool Hospital [38] also recommend blood pressure monitoring, but do not state how often this should occur.

Other recommended screening tests include regular (at least every five years) monitoring of blood glucose and serum lipids (recommended by SOMANZ [34] and South Australia [47]). Northern Territory [41] and Queensland [43] also recommend monitoring serum lipids and blood glucose, but do not state how often this should be performed. Liverpool Hospital [38] additionally recommends renal follow-up. The Royal Hospital for Women (NSW) [44] simply states that, “assessment for cardiovascular risks,” (page 7) should be performed (Table 2).

#### Prenatal counselling and prophylaxis for future pregnancies

Counselling about risk of disease recurrence in future pregnancies is recommended by Northern Territory [41], Queensland [43], Liverpool Hospital [38], Monash Health [39], and Royal Hospital for Women (NSW) [44]. SOMANZ [34] also recommends pre-pregnancy counselling for future pregnancies if risk is considered “significant” (page 30). Prophylaxis to prevent disease recurrence in future pregnancies (e.g., low-dose aspirin) is recommended by SOMANZ [34], Northern Territory [41], Queensland [43], and Royal Hospital for Women (NSW) [44].

### Risk of bias assessment

**Fig. 2:**
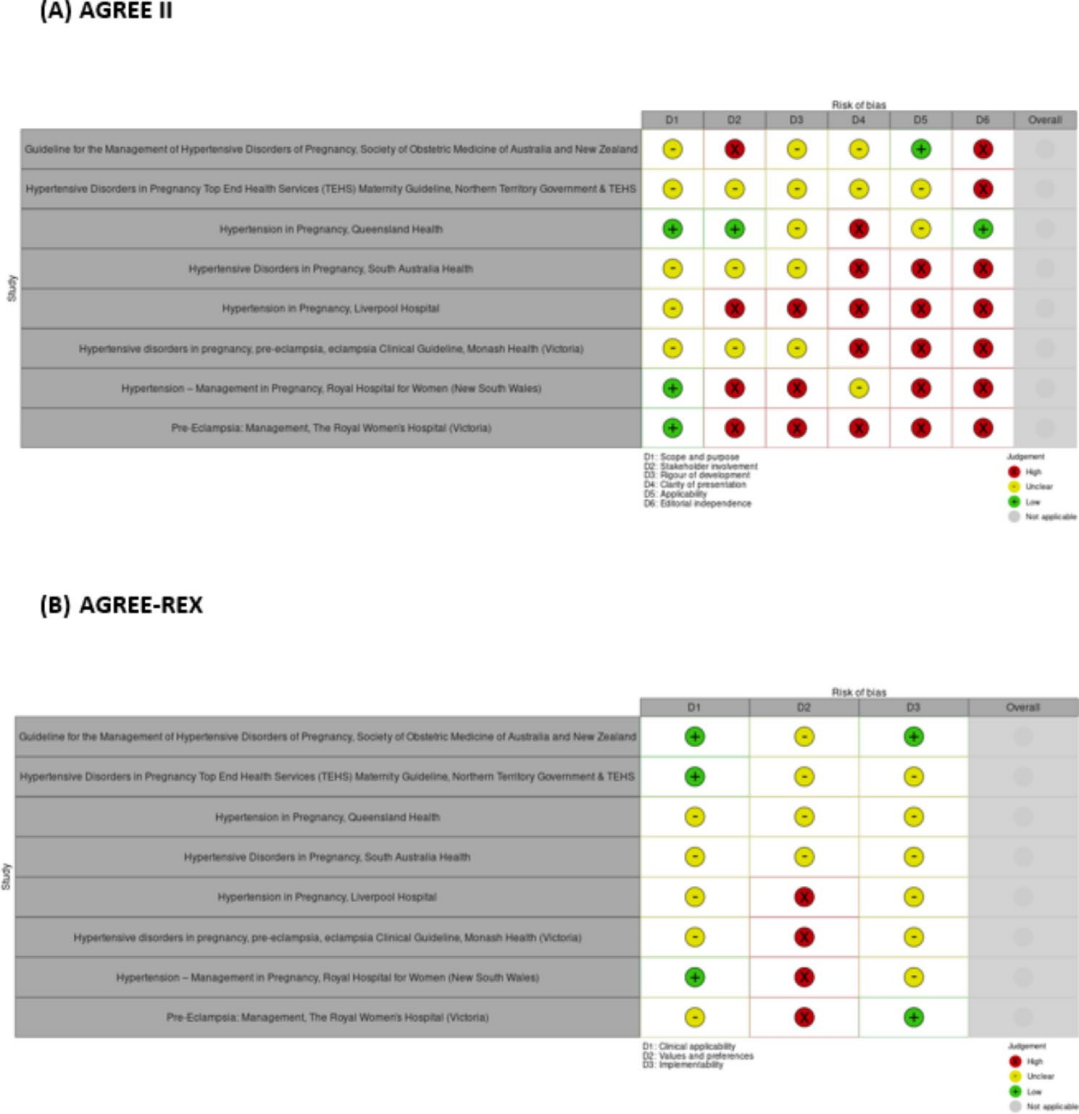
AGREE II (**A**) and AGREE REX (**B**) robvis risk of bias assessment. Adapted from McGuinness & Higgins (2020) [33]

The AGREE II tool revealed mixed results regarding guideline quality (Fig. 2, Supplementary Table 6) National and state-based guidelines were generally considered of higher quality than hospital-based guidelines. The Queensland [43] guideline was assessed to have the lowest overall risk of bias. Major risks of bias were introduced in the editorial independence and applicability domains, highlighting the difficulty that may be faced in implementing these guidelines across the country.

Assessing recommendations via AGREE-REX also revealed mixed results, with values and preferences being the most poorly reported domain (Fig. 2, Supplementary Table 7). SOMANZ [34] was found to have the most consistently high-quality recommendations.

## Discussion

Cardiovascular disease represents a significant risk to women with a history of hypertensive disorders of pregnancy. Timely counselling from their healthcare team is vital to ensure women are made aware of this risk and the early steps they can take to mitigate it. Despite this, we found that among eighteen obstetric clinical practice guidelines for the management of hypertensive disorders of pregnancy, less than half included recommendations to counsel women on their future risk of cardiovascular disease.

We considered four main aspects that should be communicated to affected women by their healthcare team, in accordance with advice from The Heart Foundation Australia [22]. These were: their increased risk of cardiovascular disease; the benefits of lifestyle intervention; the importance of regular risk factor assessment; and the risk of recurrence in future pregnancies (and associated risk-reduction strategies) [22].

We found that obstetric guidelines varied significantly in the content of their postpartum recommendations. Whilst the majority suggested that affected women should have regular risk factor screening (7/8), consensus about timing of this screening was lacking. Recommendations for prenatal counselling were also common (6/8), and some guidelines also noted the benefit of prophylaxis in future pregnancies. However, only five guidelines recommended lifestyle modification, and only four specifically recommended counselling women on their increased risk of cardiovascular disease.

We also noted significant variation in the quality of guidelines and their recommendations. We note that a significant limitation of the recommendations for management of cardiovascular disease risk was a lack of a substantial evidence base. Whilst trials are underway, existing literature does not provide clear consensus on the optimal timing of follow-up for women affected by preeclampsia or gestational hypertension, which we saw reflected in the practice recommendations [52–54]. However, the benefits of early counselling with women about lifestyle interventions and risk factor screening have become increasingly apparent, particularly in recent years [23, 24].

The current guidelines may benefit from an updated review of literature, as we note that, whilst two guidelines were published in 2021–2022 (Monash Health [39] and Queensland [43]), the majority are now at least three years old. The SOMANZ guideline [34] is now almost a decade old (published in 2014), and, given that this is the national resource and informs many of the other guidelines, it is important that this is updated to reflect the most recent evidence. We note that SOMANZ has indicated their intention to publish an updated guideline in 2023.

Guidelines generally lacked advice about tailoring their recommendations to specific groups (as assessed in AGREE REX *Item 9* – Local Application and Adoption). In the Australian context, we note the lack of specific guidance around long-term management of Aboriginal and Torres Strait Islander women. Zhao et al. (2022) highlighted the disparity in cardiovascular disease burden between First Nations and non-First Nations women in the Northern Territory, with cardiovascular disease accounting for 142.5/100,000 deaths among First Nations women and only 52.9/100,000 deaths among non-First Nations women [55]. Tailoring advice to specific groups has been proven to be an effective method to promote health behaviour change [56]. Rather than using a single set of recommendations for all Australian women, it may be beneficial to consider the use of specific recommendations to reach more vulnerable cohorts.

Whilst this review focused on content, it is also important to note the necessity of appropriate dissemination of these guidelines. A 2013 German study demonstrated that, while most clinicians were aware of the association between hypertensive disorders of pregnancy and future chronic disease, less than half were familiar with national guidelines [57]. If they were familiar with guidelines, clinicians were more likely to follow the recommended actions, such as counselling affected women [57]. These findings highlight not only the importance of appropriate practice recommendations, but also the direct dissemination of guidelines to clinicians, to ensure that guidance benefits the affected patient group. Further research into appropriate guideline dissemination in the Australian context may be warranted.

The period between a pregnancy affected by preeclampsia or gestational hypertension and the onset of cardiovascular disease provides a unique opportunity for monitoring and intervention, but only if this time is effectively utilised. Globally, obstetric guidelines are starting to recognise the importance of counselling women about their increased risk of cardiovascular disease [58, 59], and yet, less than half of Australian guidelines have followed suit. Unsurprisingly, a recent Australian survey reported that only two-thirds of practitioners discuss lifestyle adjustments with women affected by hypertensive disorders of pregnancy, and evidence has shown that many women are unaware of their increased risk of cardiovascular disease, nor do they routinely receive appropriate follow-up [14, 60].

We note that many postpartum recommendations for women are based on general, population-wide recommendations to reduce risk of cardiovascular disease. These recommendations are typically based on research conducted in older, male, or non-pregnant populations. Evidence to suggest the optimal strategy to manage cardiovascular disease risk in the postpartum population is lacking [18, 19, 61]. However, the current recommendations are broadly effective in reducing the risk of cardiovascular disease in the general population, and are therefore useful, given the lack of specific evidence tailored towards postpartum women [15–17]. It is important that targeted research into risk management among women with a history of hypertensive disorders of pregnancy be conducted to further enhance our evidence-based guidelines.

### Strengths and limitations

To our knowledge, this review is the first to explore the quality and content of published Australian clinical practice guidelines relating to cardiovascular disease following hypertensive disorders of pregnancy. This review supports the development of more uniform, robust, and evidence-based clinical practice guidelines to direct the management of long-term cardiovascular disease risk for affected women.

Our review was limited by access to publicly available guidelines only, although this is a universal limitation of systematic reviews and is not unique to this study. The guidelines included were found to be of varying quality, and many guidelines were based on secondary evidence from other guidelines. This made comparison and quality assessment more difficult, however, this limitation was accounted for in the data extraction process.

## Conclusion

The postpartum cardiovascular sequelae following hypertensive disorders of pregnancy has been well-characterised and contributes to a significant burden of disease for affected women. Among Australian clinical practice guidelines for the management of hypertensive disorders of pregnancy, only eight (44%) provided recommendations to counsel women on managing this risk, and, among these, there was considerable variation in quality and content. This review highlights the need for the development of more robust, consistent, and evidence-based guidelines to manage the risk of cardiovascular disease following preeclampsia or gestational hypertension.

### Electronic supplementary material

Below is the link to the electronic supplementary material.

**Supplementary Table 1**. Preferred Reporting Items for Systematic Reviews and Meta-Analyses (PRISMA) guidelines. **Supplementary Table 2**. OVID Medline database search strategy. **Supplementary Table 3**. EMBASE database search strategy. **Supplementary Table 4**. CINAHL database search strategy. **Supplementary Table 5**. Characteristics of included and excluded guidelines. **Supplementary Table 6**. Quality assessment - AGREE II instrument. **Supplementary Table 7**. Quality assessment - AGREE-REX instrument.

## Data Availability

All data generated or analysed during this study are included in this published article [and its supplementary information files].
