# Supplementary material for: The long-term risk of cardiovascular disease among women with a history of hypertensive disorders of pregnancy: a systematic review of clinical practice guidelines
Source: BMC Cardiovasc Disord. 2023 Sep 9;23:443. doi: 10.1186/s12872-023-03446-x (PMC10492379; doi:10.1186/s12872-023-03446-x)
Supplement: Supplementary file 1 — Supplementary Table 1. Preferred Reporting Items for Systematic Reviews and Meta-Analyses (PRISMA) guidelines. Supplementary Table 2. OVID Medline database search strategy. Supplementary Table 3. EMBASE database search strategy. Supplementary Table 4. CINAHL database search strategy. Supplementary Table 5. Characteristics of included and excluded guidelines. Supplementary Table 6. Quality assessment - AGREE II instrument. Supplementary Table 7. Quality assessment - AGREE-REX instrument. [file 12872_2023_3446_MOESM1_ESM.docx]

**Supplementary Table 1.** Preferred Reporting Items for Systematic Reviews and Meta-Analyses (PRISMA) guidelines.

| **Section and Topic** | **Item #** | **Checklist item** | **Location where item is reported** |
| --- | --- | --- | --- |
| **TITLE** | | |  |
| Title | 1 | Identify the report as a systematic review. | Page 1 |
| **ABSTRACT** | | |  |
| Abstract | 2 | See the PRISMA 2020 for Abstracts checklist. | Page 2 |
| **INTRODUCTION** | | |  |
| Rationale | 3 | Describe the rationale for the review in the context of existing knowledge. | Page 3-4 |
| Objectives | 4 | Provide an explicit statement of the objective(s) or question(s) the review addresses. | Page 3-4 |
| **METHODS** | | |  |
| Eligibility criteria | 5 | Specify the inclusion and exclusion criteria for the review and how studies were grouped for the syntheses. | Page 5 |
| Information sources | 6 | Specify all databases, registers, websites, organisations, reference lists and other sources searched or consulted to identify studies. Specify the date when each source was last searched or consulted. | Page 5 |
| Search strategy | 7 | Present the full search strategies for all databases, registers and websites, including any filters and limits used. | Table S2-S4 |
| Selection process | 8 | Specify the methods used to decide whether a study met the inclusion criteria of the review, including how many reviewers screened each record and each report retrieved, whether they worked independently, and if applicable, details of automation tools used in the process. | Page 5-6 |
| Data collection process | 9 | Specify the methods used to collect data from reports, including how many reviewers collected data from each report, whether they worked independently, any processes for obtaining or confirming data from study investigators, and if applicable, details of automation tools used in the process. | Page 5-6 |
| Data items | 10a | List and define all outcomes for which data were sought. Specify whether all results that were compatible with each outcome domain in each study were sought (e.g. for all measures, time points, analyses), and if not, the methods used to decide which results to collect. | Page 5-6 |
|  | 10b | List and define all other variables for which data were sought (e.g. participant and intervention characteristics, funding sources). Describe any assumptions made about any missing or unclear information. | Page 5-6 |
| Study risk of bias assessment | 11 | Specify the methods used to assess risk of bias in the included studies, including details of the tool(s) used, how many reviewers assessed each study and whether they worked independently, and if applicable, details of automation tools used in the process. | Page 6 |
| Effect measures | 12 | Specify for each outcome the effect measure(s) (e.g. risk ratio, mean difference) used in the synthesis or presentation of results. | Page 6 |
| Synthesis methods | 13a | Describe the processes used to decide which studies were eligible for each synthesis (e.g. tabulating the study intervention characteristics and comparing against the planned groups for each synthesis (item #5)). | Page 6 |
|  | 13b | Describe any methods required to prepare the data for presentation or synthesis, such as handling of missing summary statistics, or data conversions. | Page 6 |
|  | 13c | Describe any methods used to tabulate or visually display results of individual studies and syntheses. | Page 6 |
|  | 13d | Describe any methods used to synthesize results and provide a rationale for the choice(s). If meta-analysis was performed, describe the model(s), method(s) to identify the presence and extent of statistical heterogeneity, and software package(s) used. | Page 6 |
|  | 13e | Describe any methods used to explore possible causes of heterogeneity among study results (e.g. subgroup analysis, meta-regression). | Page 6 |
|  | 13f | Describe any sensitivity analyses conducted to assess robustness of the synthesized results. | Page 6 |
| Reporting bias assessment | 14 | Describe any methods used to assess risk of bias due to missing results in a synthesis (arising from reporting biases). | Page 6 |
| Certainty assessment | 15 | Describe any methods used to assess certainty (or confidence) in the body of evidence for an outcome. | Page 6 |
| **RESULTS** | | |  |
| Study selection | 16a | Describe the results of the search and selection process, from the number of records identified in the search to the number of studies included in the review, ideally using a flow diagram. | Figure 1 |
|  | 16b | Cite studies that might appear to meet the inclusion criteria, but which were excluded, and explain why they were excluded. | Page 7 |
| Study characteristics | 17 | Cite each included study and present its characteristics. | Page 7; Table 1 |
| Risk of bias in studies | 18 | Present assessments of risk of bias for each included study. | Figure 2 |
| Results of individual studies | 19 | For all outcomes, present, for each study: (a) summary statistics for each group (where appropriate) and (b) an effect estimate and its precision (e.g. confidence/credible interval), ideally using structured tables or plots. | N/A |
| Results of syntheses | 20a | For each synthesis, briefly summarise the characteristics and risk of bias among contributing studies. | Page 7 |
|  | 20b | Present results of all statistical syntheses conducted. If meta-analysis was done, present for each the summary estimate and its precision (e.g. confidence/credible interval) and measures of statistical heterogeneity. If comparing groups, describe the direction of the effect. | N/A |
|  | 20c | Present results of all investigations of possible causes of heterogeneity among study results. | N/A |
|  | 20d | Present results of all sensitivity analyses conducted to assess the robustness of the synthesized results. | N/A |
| Reporting biases | 21 | Present assessments of risk of bias due to missing results (arising from reporting biases) for each synthesis assessed. | Page 9-10 |
| Certainty of evidence | 22 | Present assessments of certainty (or confidence) in the body of evidence for each outcome assessed. | Page 9-10 |
| **DISCUSSION** | | |  |
| Discussion | 23a | Provide a general interpretation of the results in the context of other evidence. | Page 10-11 |
|  | 23b | Discuss any limitations of the evidence included in the review. | Page 12-13 |
|  | 23c | Discuss any limitations of the review processes used. | Page 12-13 |
|  | 23d | Discuss implications of the results for practice, policy, and future research. | Page 10-13 |
| **OTHER INFORMATION** | | |  |
| Registration and protocol | 24a | Provide registration information for the review, including register name and registration number, or state that the review was not registered. | Page 4 |
|  | 24b | Indicate where the review protocol can be accessed, or state that a protocol was not prepared. | Page 4 |
|  | 24c | Describe and explain any amendments to information provided at registration or in the protocol. | N/A |
| Support | 25 | Describe sources of financial or non-financial support for the review, and the role of the funders or sponsors in the review. | Page 14 |
| Competing interests | 26 | Declare any competing interests of review authors. | Page 14 |
| Availability of data, code and other materials | 27 | Report which of the following are publicly available and where they can be found: template data collection forms; data extracted from included studies; data used for all analyses; analytic code; any other materials used in the review. | Page 14 |

**Supplementary Table 2.** OVID Medline database search strategy.

| Search Number | Search Terms |
| --- | --- |
| 1 | exp Pregnancy/ or Pregnancy Complications/ |
| 2 | pregnan*.mp. [mp=title, abstract, original title, name of substance word, subject heading word, floating sub-heading word, keyword heading word, organism supplementary concept word, protocol supplementary concept word, rare disease supplementary concept word, unique identifier, synonyms] |
| 3 | postpartum.mp. or Postpartum Period/ |
| 4 | Postnatal Care/ |
| 5 | postnat*.mp. [mp=title, abstract, original title, name of substance word, subject heading word, floating sub-heading word, keyword heading word, organism supplementary concept word, protocol supplementary concept word, rare disease supplementary concept word, unique identifier, synonyms] |
| 6 | 1 or 2 or 3 or 4 or 5 |
| 7 | exp hypertension, pregnancy-induced/ or eclampsia/ or hellp syndrome/ or pre-eclampsia/ |
| 8 | pre?eclampsia.mp. [mp=title, abstract, original title, name of substance word, subject heading word, floating sub-heading word, keyword heading word, organism supplementary concept word, protocol supplementary concept word, rare disease supplementary concept word, unique identifier, synonyms] |
| 9 | eclampsia.mp. [mp=title, abstract, original title, name of substance word, subject heading word, floating sub-heading word, keyword heading word, organism supplementary concept word, protocol supplementary concept word, rare disease supplementary concept word, unique identifier, synonyms] |
| 10 | toxemia.mp. or Toxemia/ |
| 11 | (hypertensi* adj3 pregnan*).mp. [mp=title, abstract, original title, name of substance word, subject heading word, floating sub-heading word, keyword heading word, organism supplementary concept word, protocol supplementary concept word, rare disease supplementary concept word, unique identifier, synonyms] |
| 12 | 7 or 8 or 9 or 10 or 11 |
| 13 | 6 and 12 |
| 14 | Pregnancy Complications, Cardiovascular/ or Cardiovascular Abnormalities/ or cardiovascular.mp. or exp Cardiovascular Diseases/ or exp Cardiovascular System/ |
| 15 | heart.mp. or exp Heart Diseases/ or exp Heart/ |
| 16 | cardiac.mp. |
| 17 | exp Coronary Disease/ or coronary.mp. |
| 18 | 14 or 15 or 16 or 17 |
| 19 | 13 and 18 |
| 20 | Practice Guideline/ or Guideline/ |
| 21 | guideline*.mp. [mp=title, abstract, original title, name of substance word, subject heading word, floating sub-heading word, keyword heading word, organism supplementary concept word, protocol supplementary concept word, rare disease supplementary concept word, unique identifier, synonyms] |
| 22 | 20 or 21 |
| 23 | 19 and 22 |
| 24 | limit 23 to (yr="2012 - 2022" and english) |

**Supplementary Table 3.** EMBASE database search strategy.

| Search Number | Search Terms |
| --- | --- |
| 1 | exp pregnancy disorder/ or exp pregnancy toxemia/ or pregnancy complication/ or exp pregnancy/ |
| 2 | pregnan*.mp. [mp=title, abstract, heading word, drug trade name, original title, device manufacturer, drug manufacturer, device trade name, keyword heading word, floating subheading word, candidate term word] |
| 3 | postpartum.mp. [mp=title, abstract, heading word, drug trade name, original title, device manufacturer, drug manufacturer, device trade name, keyword heading word, floating subheading word, candidate term word] |
| 4 | postnatal care/ |
| 5 | postnat*.mp. [mp=title, abstract, heading word, drug trade name, original title, device manufacturer, drug manufacturer, device trade name, keyword heading word, floating subheading word, candidate term word] |
| 6 | 1 or 2 or 3 or 4 or 5 |
| 7 | exp "eclampsia and preeclampsia"/ or exp preeclampsia/ |
| 8 | eclampsia.mp. or exp eclampsia/ |
| 9 | pre?eclampsia.mp. [mp=title, abstract, heading word, drug trade name, original title, device manufacturer, drug manufacturer, device trade name, keyword heading word, floating subheading word, candidate term word] |
| 10 | toxemia/ or pregnancy toxemia/ or toxemia.mp. |
| 11 | (hypertensi* adj3 pregnan*).mp. [mp=title, abstract, heading word, drug trade name, original title, device manufacturer, drug manufacturer, device trade name, keyword heading word, floating subheading word, candidate term word] |
| 12 | 7 or 8 or 9 or 10 or 11 |
| 13 | 6 and 12 |
| 14 | exp cardiovascular disease/ or cariovascular.mp. |
| 15 | heart disease/ or heart/ or heart.mp. |
| 16 | cardiac.mp. |
| 17 | coronary.mp. or exp coronary artery disease/ or coronary risk/ |
| 18 | 14 or 15 or 16 or 17 |
| 19 | 13 and 18 |
| 20 | guideline.mp. or practice guideline/ |
| 21 | 19 and 20 |
| 22 | limit 21 to (english and yr="2012 - 2022") |

**Supplementary Table 4.** CINAHL database search strategy.

| Search Number | Search Terms |
| --- | --- |
| S1 | (MH “Pregnancy+”) |
| S2 | pregnan* |
| S3 | (MH “Postnatal Care+”) OR (MH “Postnatal Period+”) OR “postpartum” |
| S4 | postnat* |
| S5 | S1 OR S2 OR S3 OR S4 |
| S6 | (MH “HELLP Syndrome”) OR (MH “Eclampsia+”) OR (MH “Pre-Eclampsia+”) |
| S7 | preeclampsia |
| S8 | (MH “Toxemia”) OR “toxemia” |
| S9 | (MH “Pregnancy-Induced Hypertension+”) |
| S10 | pregnan* N3 hypertensi* |
| S11 | S6 OR S7 OR S8 OR S9 OR S10 |
| S12 | S5 AND S11 |
| S13 | (MH “Cardiovascular Diseases +”) OR (MH “Cardiovascular System+”) OR “cardiovascular” |
| S14 | (MH “Heart+”) OR “heart” |
| S15 | cardiac |
| S16 | (MH “Coronary Disease+”) OR “coronary” |
| S17 | S13 OR S14 OR S15 OR S16 |
| S18 | S13 AND S17 |
| S19 | (MH “Practice Guidelines”) |
| S20 | guideline* |
| S21 | S19 OR S20 |
| S22 | S18 AND S21 |
| S23 | S18 AND S21* |

**Supplementary Table 5.** Characteristics of included and excluded guidelines.

| **Guideline** | **Institution** | **Jurisdiction** | **Recommendations for postpartum management?** | **Included in final review?** |
| --- | --- | --- | --- | --- |
| Guideline for the management of hypertensive disorders of pregnancy (2014) | Society of Obstetric Medicine Australia and New Zealand (SOMANZ) | Multi-National (Australia and New Zealand) | Y | Y |
| Hypertensive Disorders in Pregnancy TEHD Maternity Guideline (2019) | Northern Territory Government/Top End Health Services | Territory-Based (Northern Territory) | Y | Y |
| Hypertension and Pregnancy (2021) | Queensland Health | State-Based (Queensland) | Y | Y |
| Hypertensive Disorders of Pregnancy (2020) | South Australia Health | State-Based (South Australia) | Y | Y |
| Hypertension in Pregnancy (2018) | Liverpool Hospital (NSW) | Hospital-Based (Liverpool Hospital, New South Wales) | Y | Y |
| Hypertensive disorders in pregnancy, pre-eclampsia, eclampsia clinical guideline (2022) | Monash Health (VIC) | Hospital-Based (Monash Health, Victoria) | Y | Y |
| Hypertension – Management in pregnancy (2020) | Royal Hospital for Women (NSW) | Hospital-Based (Royal Hospital for Women, New South Wales) | Y | Y |
| Pre-Eclampsia: Management (2020) | The Royal Women’s Hospital (VIC) | Hospital Based (The Royal Women’s Hospital, Victoria) | Y | Y |
| Hypertension in pregnancy (2017) | Canberra Hospital and Health Services | Territory-Based (Australian Capital Territory) | N | N |
| Hypertension in pregnancy: Midwifery care (2018) | Government of Western Australia, North Metropolitan Health Service | Hospital-Based (King Edward Memorial Hospital, Western Australia) | N | N |
| Hypertension in pregnancy: Medical management (2020) | Government of Western Australia, North Metropolitan Health Service | Hospital-Based (King Edward Memorial Hospital, Western Australia) | N | N |
| Hypertension in pregnancy (Pre-eclampsia & eclampsia) (2019) | Peninsula Health (VIC) | Hospital-Based (Peninsula Health, Victoria) | N | N |
| Clinical practice guidelines: Obstetrics/pre-eclampsia (2016) | Queensland Ambulance Service | State-Based (Queensland) | N | N |
| Pre-eclampsia – Intrapartum care (2020) | Royal Hospital for Women (NSW) | Hospital-Based (Royal Hospital for Women, New South Wales) | N | N |
| Severe and/or urgent hypertension in pregnancy (2020) | Royal Hospital for Women (NSW) | Hospital-Based (Royal Hospital for Women, New South Wales) | N | N |
| Hypertension in pregnancy (2018) | Safer Care Victoria/Victorian Agency for Health Information | State-Based (Victoria) | N | N |
| Hypertension in pregnancy (2021) | The Royal Children’s Hospital Melbourne | Hospital-Based (The Royal Children’s Hospital, Victoria) | N | N |
| Hypertension – management of acute (2020) | The Royal Women’s Hospital (VIC) | Hospital Based (The Royal Women’s Hospital, Victoria) | N | N |

| Title | Guideline for the Management of Hypertensive Disorders of Pregnancy | Hypertensive Disorders in Pregnancy TEHS Maternity Guideline | Hypertension and Pregnancy | Hypertensive Disorders in Pregnancy | Hypertension in Pregnancy | Hypertensive disorders in pregnancy, pre-eclampsia, eclampsia Clinical Guideline | Hypertension - Management in Pregnancy | Pre-Eclampsia: Management |
| --- | --- | --- | --- | --- | --- | --- | --- | --- |
| Institution | SOMANZ | Northern Territory Government/Top End Health Services | Queensland Health | South Australia Health | Liverpool Hospital (NSW) | Monash Health (VIC) | Royal Hospital for Women (NSW) | The Royal Women's Hospital (VIC) |
| Domain 1 | 39 | 44 | 100 | 50 | 56 | 50 | 83 | 78 |
| Domain 2 | 6 | 33 | 94 | 50 | 0 | 33 | 28 | 28 |
| Domain 3 | 42 | 38 | 52 | 48 | 23 | 65 | 23 | 10 |
| Domain 4 | 56 | 61 | 83 | 100 | 83 | 100 | 67 | 89 |
| Domain 5 | 71 | 42 | 63 | 21 | 22 | 25 | 21 | 29 |
| Domain 6 | 8 | 0 | 75 | 0 | 0 | 0 | 0 | 0 |

**Supplementary Table 6.** Quality assessment – AGREE II instrument.

Domain 1: Scope and purpose

Domain 2: Stakeholder involvement

Domain 3: Rigour of development

Domain 4: Clarity of presentation

Domain 5: Applicability

Domain 6: Editorial independence

**Supplementary Table 7.** Quality assessment – AGREE-REX instrument.

| Title | Guideline for the Management of Hypertensive Disorders of Pregnancy | Hypertensive Disorders in Pregnancy TEHS Maternity Guideline | Hypertension and Pregnancy | Hypertensive Disorders in Pregnancy | Hypertension in Pregnancy | Hypertensive disorders in pregnancy, pre-eclampsia, eclampsia Clinical Guideline | Hypertension - Management in Pregnancy | Pre-Eclampsia: Management |
| --- | --- | --- | --- | --- | --- | --- | --- | --- |
| Institution | SOMANZ | Northern Territory Government/Top End Health Services | Queensland Health | South Australia Health | Liverpool Hospital (NSW) | Monash Health (VIC) | Royal Hospital for Women (NSW) | The Royal Women's Hospital (VIC) |
| Domain 1 | 83 | 78 | 61 | 39 | 44 | 33 | 72 | 56 |
| Domain 2 | 50 | 61 | 61 | 33 | 11 | 11 | 22 | 28 |
| Domain 3 | 83 | 50 | 50 | 67 | 58 | 58 | 58 | 75 |

Domain 1: Clinical applicability

Domain 2: Values and preferences

Domain 3: Implementability
